# Special Regulation of *GhANT* in Ovules Increases the Size of Cotton Seeds

**DOI:** 10.3390/genes16080912

**Published:** 2025-07-30

**Authors:** Ning Liu, Yuping Chen, Yangbing Guan, Geyi Guan, Jian Yang, Feng Nie, Kui Ming, Wenqin Bai, Ming Luo, Xingying Yan

**Affiliations:** 1 College of Agronomy and Biotechnology, Southwest University, Chongqing 400715, China; 2 Integrative Science Center of Germplasm Creation in Western China (CHONGQING) Science City, College of Agronomy and Biotechnology, Southwest University, Beibei, Chongqing 400715, China

**Keywords:** *GhANT*, cotton, ovule, seeds size, seed yield

## Abstract

Background: *Gossypium hirsutum* L. is one of the main economic crops worldwide, and increasing the size/weight of its seeds is a potential strategy to improve its seed-related yield. AINTEGUMENTA (ANT) is an organogenesis transcription factor mediating cell proliferation and expansion in *Arabidopsis*, but little is known about its candidate function in upland cotton seed. Results: In this study, functional characterization of *GhANT* in the cotton seed development stage was performed. The expression pattern analysis showed that *GhANT* was predominantly expressed in the ovules, and its expression was consistent with the ovules’ development stage. Heterologous expression of *GhANT* in *Arabidopsis* promoted plant organ growth and led to larger seeds. Importantly, specific expression of *GhANT* by the TFM7 promoter in the cotton ovules enlarged the seeds and increased the cotton seed yield, as compared with the wild-type in a three-year field trial. Furthermore, transcription level analysis showed that numerous genes involved in cell division were up-regulated in the ovules of TFM7::*GhANT* lines in comparison to the wild-type. These results indicate that *GhANT* is a potential genetic resource for improving cotton seed yield through its molecular links with cell cycle controllers.

## 1. Introduction

Seed size is an essential agronomic trait considered in the domestication and breeding of many crops. Increasing the seed size can drive seed yield improvements under conditions of a fixed seed number per plant. Cotton is one of the most important sources of superior natural fiber, and its seeds are rich in oils and proteins. The yields of cotton fiber and seeds are important agricultural traits of upland cotton. The cotton fiber quality is judged from such indicators as the fiber strength, length and micronaire value, but these are negatively correlated with the boll weight and seed size [1]. Our previous report showed that spatiotemporal regulation of auxin biosynthesis in cotton significantly improved the quality of the fiber, but the seeds were smaller [2]. This inspired us to create a new genetic resource with larger seeds but without a decrease in the quality or quantity of fiber; larger seeds can improve the ultimate “sink strength” of crops and are an important way of improving plant growth and tolerance to certain abiotic stresses [3].

More and more pathways for the seed size regulatory network have been revealed, including the HAIKU (IKU) pathway [4], ubiquitin–proteasome pathway [5], G (Guanosine triphosphate) protein regulatory pathway [6], mitogen-activated protein kinase (MAPK) pathway [7], transcriptional regulators pathway [8], and phytohormone regulatory pathways, including the auxin [9], brassinosteroid (BR) [10], gibberellin (GA) [11], jasmonic acid (JA) [12], cytokinin (CK) [13], abscisic acid (ABA) [14], and microRNA (miRNA) regulatory pathways.

AINTEGUMENTA (ANT) is a member of the AP2 (APETALA2)/EREBP (ethylene response element binding protein) family related to cell proliferation and organ growth. ANT is induced by ARGOS, a protein with a function in auxin signaling. Loss-of-function ARGOS or ANT mutants exhibited smaller leaves due to premature proliferation arrest and reduced cell numbers [15,16]. AtANT mutants with fewer and smaller flowers are sterile; this is attributed to the absence of female gametophytes and integuments, such that ovule development is disordered and retarded [17]. Ectopic expression of the 35S::ANT transgene enlarges embryonic and shoot organs (seeds, leaves, flowers, and root systems) with cell division in the integuments during ovule development, but most 35S::ANT transgenic plants show female and male sterility due to abnormally extended proliferation of the chalazal nucellular cells or a failure of the anthers to dehisce [18]. In *Arabidopsis*, AtANT is involved in regulating the cycle of cell division by CYCLIN D3;1 (CYCD3;1) to maintain the meristematic competence of cells during organogenesis, and overexpression of CYCD3;1 increases the cell number in the leaves [19]. The D-type cyclins (CYCDs) promote the transition from the G1 to S phases of the mitotic cell cycle. However, there is no direct interaction between the promoter sequences of AtCycD and AtANT, and it has been speculated that AtANT and AtCycD3 operate independently in regulating organ growth [20]. Recently, 200 potential ANT target genes, involved in functions such as polarity specification, floral organ development, meristem development, auxin signaling and several genes associated with lateral organ growth [21]. 

To explore whether GhANT has the function of enhancing seed size, we generated transgenic *Arabidopsis* expressing GhANT driven by cauliflower mosaic virus (CaMV) 35S promoter and transgenic cotton expressing GhANT controlled by an ovule special promoter, pTFM7 [22,23,24]. The ectopic expression of GhANT caused significant alterations in seed size in *Arabidopsis*. Moreover, molecular biology experiments and field trials showed that *GhANT* overexpression increased the seed size, as compared to the wild-type in cotton. Transcriptome sequencing data and qRT-PCR showed that GhANT promoted the expression of cyclin genes, and a dual-luciferase reporter assay provided evidence that GhANT could coordinate the cell number by regulating cell cycle control. These results indicate the important role of GhANT in regulating seed size in cotton.

## 2. Materials and Methods

### 2.1. Sequence Analysis

The GhANT sequence was identified via the BLASTp program using the AtANT protein sequence (AT4G37750) and obtained according to *Gossypium arboreum* AP2-like ethylene-responsive transcription factor ANT (Ghi_A06G01316). Other full-length ANT protein sequences were obtained from the cottonMD (https://yanglab.hzau.edu.cn/CottonMD/blast.1) (accessed on 29 July 2005). The sequences were aligned using ClustalW, and phylogenetic trees were constructed via the neighbor-joining method (NJ) using the MEGA11 (Molecular Evolutionary Genetics Analysis) program [14]. The percentages of replicate trees in which the associated taxa clustered together in the bootstrap test (1000 replicates) are shown next to the branches. The numbers on the tree branches indicate bootstrap probability values.

### 2.2. RNA Extraction and Quantitative RT-PCR Analyses

In order to analyze the expression characteristics of GhANT, flowers were labeled as 0 days post-anthesis (DPA) on the day of flowering. Samples were collected from ovules and fibers at different developmental stages (0~25 DPA) RNA was extracted using a rapid plant RNA extraction kit (Aidlab, Beijing, China). The petals, stamens, pistils, young stems (3rd internode), and bolls (18 DPA) were collected from wild-type cotton at 0 DPA. The fourth main-stem leaves from the apex (functional leaves) were taken at 0 DPA.

In order to analyze the activity of the TFM7 promoter, the ovules of (0~25 DPA) of pTFM7::GUS transgenetics were collected, and the transcript levels of GUS were analyzed by qPCR-GUS primer (Supplementary Table S1).

Single-stranded cDNA was synthesized from the total RNA using a cDNA synthesis kit (TaKaRa, Dalian, China). The gene-specific primers used for real-time PCR amplification were *GhANT*RT-up (5′-ACGAGGTACGATGTGGAACG-3′) and *GhANT*RT-dn (5′-TCCAGTCTGGTTGAGACCCA-3′). The cotton histon3 gene (AF024716) was amplified as an internal standard. The target gene primers are provided in Supplementary Table S1. Quantitative real-time RT-PCR (qRT-PCR) was performed on a CFX96 real-time PCR detection system with SYBR Green supermix (Bio-Rad, Hercules, CA, USA). Three independent biological samples were used in the qRT-PCR. The reactions were duplicated 3 times, and the data were analyzed using the Bio-Rad CFX Manager 2.0 software provided by the manufacturer.

### 2.3. Plasmid Construction and Plant Transformation

The GhANT gene was amplified from upland cotton cDNA using a forward primer (5′-ATGAAGTCCATGAGCAATGA-3′) and a reverse primer (5′-ACTGCCTGGACAGATGCTTAG-3′) and cloned into the TA cloning vector pMD19-T (TaKaRa, Dalian, China). To construct an overexpression vector of GhANT (35S::GhANT), the CaMV 35S promoter was used instead of the intrinsic promoter. The product of the polymerase chain reaction (PCR) was cloned into the binary vector pLGN using the Xbal I and Sal I restriction sites. The pLGN vector was reformed on the basis of the p5 vector [25], and a LoxpFRT recombinase recognition site was added, constructed on both ends of the expression box. The constructed vector was used to transform *Arabidopsis* plants using the planta Agrobacterium-mediated method, and the transgenic seedlings were selected using 1/2 MS agarose plates containing 50 mg/mL kanamycin sulfate. Homozygous transgenic plants were used for further analyses.

The GhTFM7 promoter was amplified from cotton genomic DNA using the pTFM7 primers (Table S1), and integrated the sequence into pLGN with modification (containing GhTFM7 promoter::nos and 35S promoter::GUS:NPTII::nos fusion genes) to construct GhTFM7 promoter vectors (pTFM7::GUS). To construct specific expression vector of cotton GhANT (TFM7::GhANT), the CaMV 35S promoter in the expression vector (35S::GhANT) was replaced with a TFM7 promoter. The TFM7 promoter was inserted into the pLGN vector and digested with SalI and BamHI enzymes to generate the vector pLGN-TFM7::GhANT. Transgenic plants were generated via agrobacterium-mediated transformation [25]. Kanamycin-resistant and GUS-positive plants in generation T0 were screened out for further study.

### 2.4. Transient Expression in Tobacco Epidermal Cells and Microscopy Observation

GFP proteins were fused at the C-termini of GhANT’s coding sequences (without the stop codon) in binary vector pCAMBIA2300 using BamHI and kpnI to construct vector (GhANT::eGFP). The agrobacterium was resuspended and diluted with infiltration buffer (10 mM MgCl_2_ and 100 μM acetosyringone) to OD600 (0.01 to 0.05), then infiltrated into eight-week-old *Nicotiana benthamiana* (*N. benthamiana)* leaves for transient expression. After three days of growth at 25 °C, the infiltrated leaves were harvested for analysis. The fluorescence signal was observed using a Leica SP8 confocal microscope equipped with a HyD detector under a 40× oil immersion objective lens. The acquisition parameters were as follows: GFP, excitation at 488 nm, emission from 496 to 530 nm; DAPI (4′,6-diamidino-2-phenylindole), excitation at 405 nm, emission from 420 to 464 nm.

### 2.5. Growth Conditions and Phenotype Analysis

Through an expression analysis of GhANT genes in transgenic cotton ovules, 12 homologous transgenic lines were obtained by means of self-crossing, and their performance was compared with a WT line (Jimian No. 14) grown in parallel in the field. To assess the agronomic performance of the transgenic lines, generation T2, T3, and T4 lines of TFM7::GhANT were planted on an experimental farm at Southwest University (Chongqing, China) in 10 plants per row. The plants were grown in field conditions for a randomized comparative trial with three replications. Each block was 18 m^2^ (4 m × 4.5 m).

The boll number was counted at 130 DPA. After harvest, the fibers and seeds were weighed separately, and the following parameters were determined: boll weight, number of seeds per boll, lint index, lint percentage (fiber weight/seed cotton weight), and seed index (the weight of 100 seeds). Fiber from each line was sampled randomly and sent to the National Center for Evaluation of Fiber Quality (Anyang, China) to measure its quality.

To compare the seed size of 35S::GhANT transgenetic line in *Arabidopsis* with WT, the mature seed was observed by ZEISS SteREO Discovery.V20 (ZEISS, Oberkochen, Germany). More than ten seeds’ length and width from an independent transgenetic line were measured by Image J 1.53e (National Institute of Health, Bethesda, MD, USA). To compare the seed cell size and number, more than ten -1 DPA ovule cells from the wild-type and GhANT-1 were examined via scanning electron microscopy (SEM) (Hitach SN3400, Hitachi, Tokyo, Japan).

### 2.6. Southern Blot and mRNA In Situ Hybridization

For the genomic Southern blot analysis, total DNA extracted from WT and TFM7::GhANT cotton was digested with the restriction enzyme HindIII, because there were no HindIII sites within the cloned GhANT cDNA sequence. An NPTII fragment amplified from the vector p5 served as the probe; gene-specific primers are listed in Supplementary Table S1. DIG High Prime DNA Labeling and Detection Starter Kit II (Roche, Mannheim, Germany) was employed to prepare digoxigenin (Dig)-labeled probes and to detect hybridization signals.

To study the expression patterns of *GhANT* in cotton ovule organ development in detail, an in situ hybridization analysis was conducted using ovules from 2 DPA, at which point the ovules are initiated and develop. In situ hybridization was performed essentially as described by Zeng et al. 2019 [26]. The DNA template of the probe was amplified from the cDNA of *GhANT* using sense primers and anti-primers (Supplementary Table S1). Paraffin sections of the ovules were deparaffinized and incubated with a Dig-labelled RNA probe. Then, the sections were incubated with anti-Dig-AP conjugate (Roche, Indianapolis, IN, USA), and the signal was detected using NBT/BCIP solution (Roche, Indianapolis, IN, USA). Sections incubated with the sense RNA probe were used as the negative control. Images were captured using a microscope (Olympus, Tokyo, Japan). SP6 and T7 RNA polymerase were used as the sense and antisense probes, respectively, in the PCR amplification.

### 2.7. RNA-Sequencing

The 2-DPA ovules from pTFM7::GhANT line 1 and WT were collected to construct RNA-seq libraries. The library construction, sequencing platform, data processing, and analysis were conducted by Majorbio Bioinformatics Technology Co., Ltd. (Shanghai, China). Genes that met the criteria of an adjusted *p*-value < 0.05 and |log_2_(-FoldChange)| > 1 were considered to be differentially expressed genes (DEGs). This analysis was conducted to explore the changes in gene expression and the potential biological pathways involved in the ovule development process of special-regulated transgenic cotton lines. The data were visualized using TBtools software (v0.67).

### 2.8. Dual-Luciferase Reporter Assay

The dual-luciferase reporter assay was performed as described previously [27]. The promoter sequences of *Gh_D05G2257 (Cyclin-A1-1-like protein) and Gh_A02G1079 (Cyclin-A1-1-like protein)* were cloned into pGreenII 0800-LUC, and the coding region of GhANT was cloned into a pCAMBIA2300-eGFP vector. Dual-luciferase reporter activities were quantified using the Dual-Glo Luciferase Assay System (Promega E2940, Promega, Madison, WI, USA).

### 2.9. Statistical Analysis

The data from the qRT-PCR analysis, the seed size measurement, and the dual-luciferase reporter system were analyzed using Student’s *t*-tests. The field trials were analyzed using one-way ANOVA with Tukey’s multiple-comparison test. Each experiment was repeated at least three times, and error bars represent means ± SD (*n* = 3). The number (n) of biological replicates is indicated in each legend or methods. The above statistical analyses were performed with GraphPad Prism (v.9.0).

## 3. Results

### 3.1. Characteristics of the GhANT Gene in Cotton

**Figure 1:**
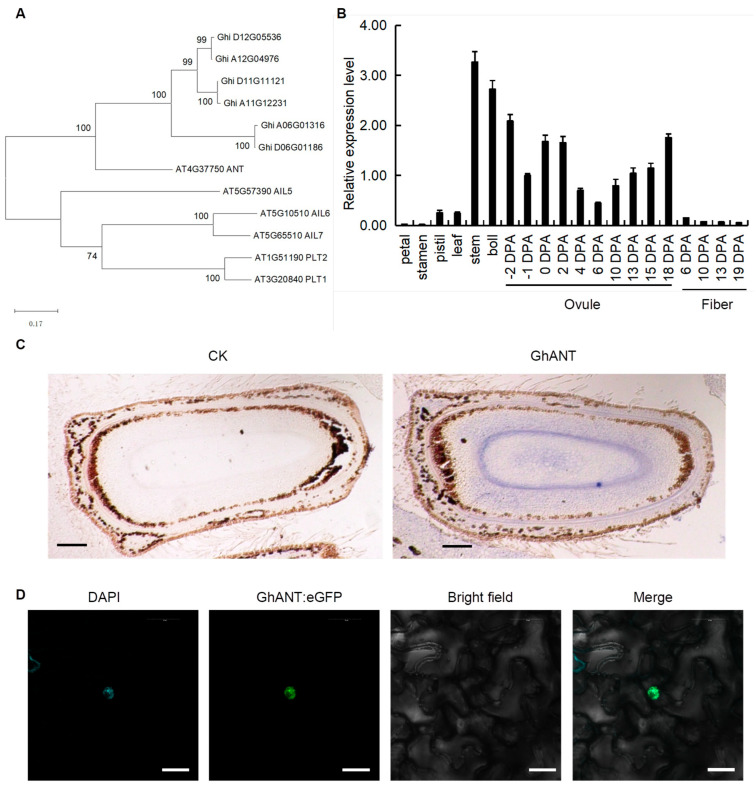
Cloning and characterization of *GhANT*. (**A**) A phylogenetic analysis of AINTEGUMENTA proteins from *Gossypium hirsutum (G. hirsutum)* and *Arabidopsis*. The numbers beside each node represent bootstrap values based on 1000 replications, from full-length protein alignment using ClustalW. The scale bar indicates the relative amount of change along the branches. (**B**) The expression of *GhANT* in various organs and different developmental stages of ovules and fibers. Error bars indicate the SD of three replicates. (**C**) The in situ hybridization pattern of *GhANT* mRNA. Hybridization of a wild-type inflorescence with a *GhANT* sense probe. Bar = 50 µm. (**D**) The subcellular localization of GhANT::eGFP in *N. benthamiana* leaves. 35S::GhANT-eGFP fluorescence; DAPI: 4′,6-Diamidino-2-phenylindole dihydrochloride, fluorescent stain of nuclei; Merge: merged images of BF (Bright Field), eGFP, and DAPI; scale bar, 10 μm.

The results showed that the mRNA sequence of *GhANT* was 2043 bp in length and encoded a protein comprising 681 amino acids. The phylogenetic analysis revealed that GhANT has high sequence homology to AtANT (AT4G37750), sharing 83% amino acid sequence identity (Figure 1A). To decipher the biological function of *GhANT* in cotton, its expression patterns were examined by means of qRT-PCR in wild-type cotton plants grown under normal conditions. *GhANT* was expressed in all detected tissues: petals, stamens, pistils, young stems (third internode), bolls (18 DPA), and the fourth main-stem leaves from the apex (functional leaves). The highest expression level of the *GhANT* gene was found in young stems, and its transcript was also detected in bolls and ovules. *GhANT* was expressed continuously with the ovules’ development, while its transcriptional levels decreased gradually throughout the process of fiber development (Figure 1B). In situ hybridization showed that *GhANT* mRNAs were enriched in the 2DPA ovules (Figure 1C). This means that GhANT may have an important function in ovule development. To verify the protein localization, these GhANT genes were then fused with green fluorescent protein (GFP) at their C-termini. A noticeable GhANT:GFP signal was found in the nucleus (Figure 1D), suggesting its possible role in regulating gene expression.

### 3.2. Transgenic Arabidopsis Plants Expressing GhANT Exhibit Larger Organs

**Figure 2:**
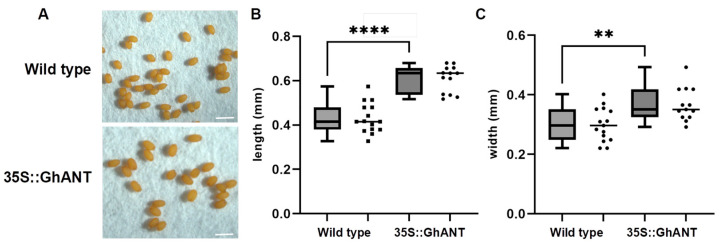
Phenotype analyses of 35S::*GhANT* in *Arabidopsis*. (**A**) Seed phenotypes of WT and 35S::*GhANT* transgenic *Arabidopsis,* Bar = 1 mm. (**B**,**C**) Seed length and width of WT and 35S::*GhANT* transgenic plants. Each sample was repeated 10 times. Statistical analysis was performed by two-sided Student’s *t*-tests. The data are the mean ± s.d. *n* ≥ 10 independent biological samples. Statistical analysis was performed by two-sided Student’s *t*-tests (**, *p* < 0.01;****, *p* < 0.0001). (**B**,**C**) repeated three times with similar results.

To explore the potential function of GhANT, wild-type *Arabidopsis* (Col-0) plants were transformed with *GhANT* under the control of the CaMV 35S promoter (PLGN-35S::*GhANT*). Our observations indicate that heterologous expression of the GhANT gene affects the growth and development of *Arabidopsis thaliana* (Figure S1A–C). A further analysis revealed that the seed length and width of the transgenic *Arabidopsis* lines increased significantly compared to the wild-type (Figure 2A–C). This suggests that this gene has a certain impact on the size of *Arabidopsis* seeds.

### 3.3. Up-Regulation of GhANT by TFM7 Promoter in Cotton Ovules

**Figure 3:**
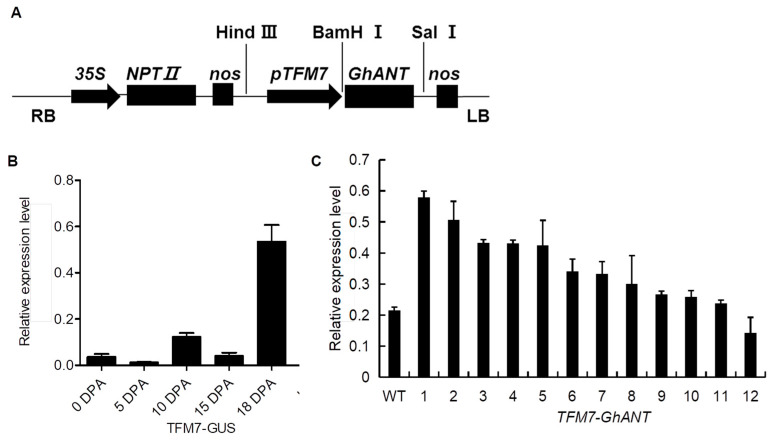
A molecular analysis of relative expression levels in cotton plants. (**A**) The structure of TFM7::GhANT. npt II, neomycin phosphotransferase gene; 35S, cauliflower mosaic virus 35S promoter. (**B**) GUS expression in tissues of *TFM7::GUS* cotton under greenhouse conditions. Error bars indicate the SD of three replicates. (**C**) A qRT-PCR analysis of the expression of *GhANT* in a wild-type line and 12 independent T1 transgenic lines (*TFM7::GhANT*). Error bars indicate the SD of three replicates.

To investigate the possible role of GhANT in ovule development, a fruit-specific promoter (*TFM7*) was used to construct a vector (Figure 3A), and transgenic cotton plants with p*TFM7::GUS* and p*TFM7::GhANT* were generated. Specific detection of the TFM7 promoter showed that *GUS* driven by the TFM7 promoter was expressed in developing ovules as expected, and the transcript level reached its peak in ovules at 18 DPA (Figure 3B), suggesting that TFM7 can specifically promote gene expression in ovules. Similarly, *GhANT* showed predominant expression in 18 DPA ovules in the *GhANT* transgenic lines (Figure S2). *pTFM7::GhANT line 1* (*GhANT-1*) and *pTFM7::GhANT line 2* (*GhANT-2*) showed higher expression levels of *GhANT* than other *pTFM7::GhANT* transformants in 18 DPA ovules and, therefore, were selected for further investigation (Figure 3C). A Southern blotting analysis showed that the *GhANT-1* plants harbored a single copy of the transgenic cassette, while *GhANT-2* plants contained a double copy of the integrated cassette (Figure S3). When the fruit-specific promoter *TFM7* was used to control the expression of *GhANT*, there was no obvious change in the growth and development of vegetative organs (Figure S4).

### 3.4. Specific Up-Regulation of GhANT in Ovules Increases Seed Size and Improves Cotton Yield

**Figure 4:**
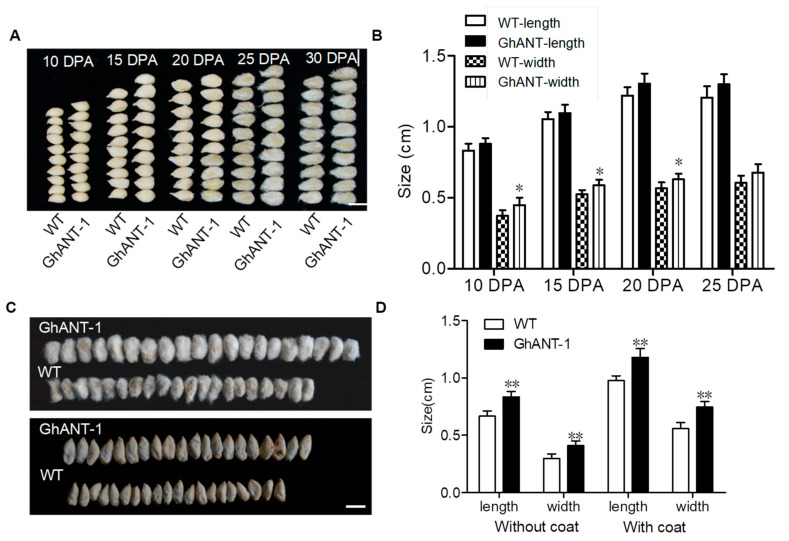
A comparison of seed sizes between WT and *pTFM7::GhANT*. (**A**) Phenotypes of seeds in different development stages of transgenic cotton and WT. Scale = 1 cm. Each sample was repeated 5 times. (**B**) Statistics of seed length and width in different development stages of transgenic cotton and WT. Statistical analysis was performed by two-sided Student’s *t*-tests. The data are the mean ± s.d. *n* = 3 independent biological experiments. (**C**) The sizes of mature seeds with and without coats. Scale = 1 cm. (**D**) Statistic of seed sizes with and without coats. Statistical analysis was performed by two-sided Student’s *t*-tests (*, *p* < 0.05; **, *p* < 0.01). The data are the mean ± s.d. *n* = 3 independent biological experiments.

The size of the ovules is a main factor in the final yield. Ovule length and width of cotton seeds at 10, 15, 20, 25, and 30 DPA in *TFM7::GhANT* cotton were increased significantly compared to those for the WT (Figure 4A,B), and the size of the seeds did not change significantly after 25 DPA. The mature seed size with or without a coat was measured for generation T2, and the results showed that *GhANT-1* enhances the seed size with or without coats (Figure 4C,D).

Ovules play a central role in plant reproduction. In order to further assess the performance of the up-regulated *GhANT* cotton in terms of yield improvement, we selected *GhANT-1* and *GhANT-2* (generations T3, T4, and T5) for field trials. After harvest, the average boll number and the seed number per boll of the transgenic lines showed no significant changes when compared to the WT (Table 1). These results suggest that the increase in seed size with up-regulated *GhANT* is not due to a reduction in seed number. Further, statistics showed that the transgenic lines produced statistically higher seed index values and boll weights than the WT plants in all three years of the field trials (Table 1). In addition, the fiber quality indicators of *GhANT-1* and *GhANT-2* in the field trial, compared to the wild-type control, such as their micronaire values, were not changed, demonstrating that there was no negative influence on the quality of transgenic fibers, except for enhancing the strength in the T2 generation.

### 3.5. Transcriptome Analysis of TFM7-GhANT and WT Plants

**Figure 5:**
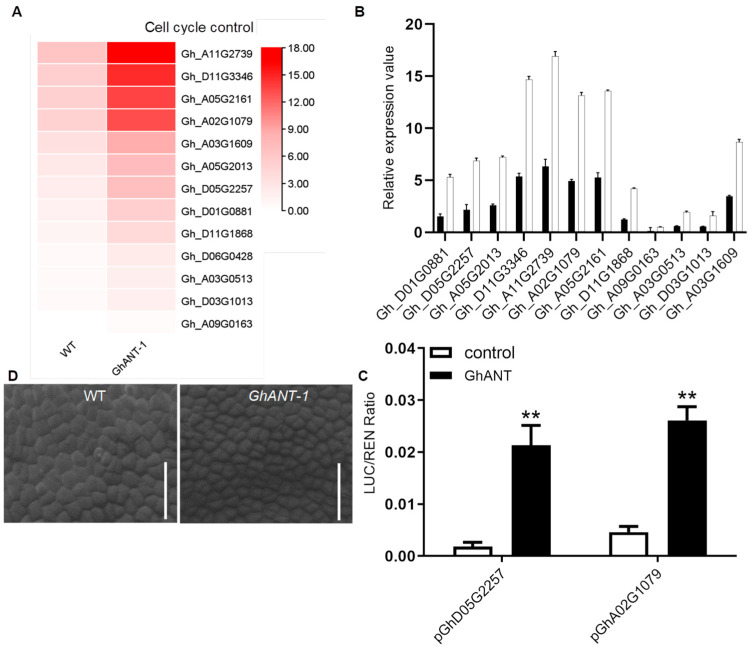
(**A**) A heatmap showing the expression pattern of genes involved in the cyclin protein. (**B**) qRT-PCR validation of the expression of 12 DEGs from the RNA-Seq analyses. The data are the mean ± s.d. *n* = 3 independent biological experiments. (**C**) Epidermal cells (×150) from -1 DPA ovules from the wild-type and *GhANT-1*, observed via SEM, bar = 20 μm. (**D**) The transactivation activity of Gh_D05G2257 and Gh_A02G1079 promoters by GhANT was calculated based on the ratio LUC/REN. Error bars indicate SD of three biological replicates. Asterisks represent significant differenceas compared with wild type were determined by Student’s *t*-test (** *p* < 0.01).

To investigate the genome-wide effects of *GhANT* on transcription, we performed RNA sequencing to analyze the global transcriptional profiles using age-matched 18 DPA ovules. For comparative analysis, publicly available transcriptomic sequence data were mined. Among the 1652 differentially expressed genes (DEGs) with two-fold or greater alteration, 729 were up-regulated and 923 were down-regulated in the transgenic line. These DEGs could be assigned to different families, such as 12 to fructose and mannose metabolism, 20 to glycolysis/gluconeogenesis, and 13 to cell cycle control. An enrichment analysis of the DEGs identified “cell cycle control” as a top annotated pathway. Additionally, 13 DEGs were selected for further verification via qRT-PCR, including cell cycle-related cotton genes, and all selected genes exhibited the same expression tendency as shown in the original data (Figure 5A,B). We were thus curious to know whether cyclin protein-coding genes are direct targets of GhANT. A transient expression analysis in tobacco showed that the LUC/REN ratios in leaves carrying both Gh_D05G2257 and Gh_A02G1079 effector and the *GhANT* reporter were significantly greater than those in leaves carrying GhANT alone. These observations showed that GhANT could activate the expression of Gh_D05G2257 and Gh_A02G1079 (Figure 5C).

A change in organ size can reflect an alteration in the size or number of cells, or both. To evaluate the possibility that *ANT* might be involved in ovule development, we observed the epidermis cells of -1 DPA ovules in the wild-type and *GhANT -1* via scanning electron microscopy. No obvious differences in cell size were detected in the epidermis cells, but the cell numbers of *GhANT-1* lines were much greater than those of the wild-type (Figure 5D).

## 4. Discussion

ANT plays an important role in mosses [28] and dicots such as *A. majus* [29,30] and *B. napus* [31]; in monocots such as *A. crassa* [32], *Tobacco* [33], and *Petunia* [34]; in gymnosperms such as *P. thunbergii* [35] and so on. From this point of view, we were curious as to the function of ANT in cotton.

Based on a previous report focusing on the model plant *Arabidopsis*, we recovered one putative ortholog. A sequence alignment with homologous ANT proteins identified the characteristic and highly conserved AP2 motif, which represents the recognition site for ANT. A phylogenetic analysis suggested that GhANT is a putative ortholog of AtANT. The functions of ANT in plant development are reflected in the distribution of its mRNA: ANT transcripts can be detected not only in the flower, but also in young growing organs and developing fruit [36]. Overexpression of ANT causes enlarged embryos and lateral organs in *A. thaliana*. In this study, the seed was increased in 35S::GhANT transgenic lines, suggesting that GhANT positively regulates seed development (Figure 2). This result is consistent with the function of the AtANT gene in the leaves and floral organs and suggest that GhANT can be exploited for crop improvements, such as increasing seed sizes.

The up-regulation of GhANT in cotton via 35S may cause sterility. To address this, we used TFM7 promoter to enhance the expression of GhANT in cotton. In a previous study, TFM7 showed a special expression pattern in fruit [22,23,24]. We obtained 12 lines of TFM7::GhANT transgenic cotton plants, among which GhANT-1 and GhANT-2 had the highest expression levels of GhANT (Figure 3B), and their seed size was larger than that of the WT (Figure 4). We compared the agronomic performance of transgenic lines GhANT-1 and GhANT-2 with that of the wild-type control in field trials. In the three-year test, the yield components, including the seed weight per boll and seed index, of the transgenic line were higher than those of the wild-type, possibly because the arrangement of seeds is more compact. Bigger seeds mean a larger area of the seed coat, bearing more fibers. Thus, no obvious change was observed in the lint index or lint percentage. Additionally, no significant changes in plant growth, yield traits, and fiber lengths, micronaire values, except for enhancing the fiber strength (T2), were found between the transgenic lines and the wild-type. Thus, the field trial results confirmed that special up-regulation of GhANT enhanced the seed yield (Table 1).

Ovules play a pivotal role in the reproductive life cycle of plants. How does GhANT control the cell number during organogenesis? Based on our results, we can speculate as to the possible mechanisms by which ANT regulates the cell number and size of mature organs. To test this idea, we observed the cell numbers and cell sizes of -1DPA ovule epidermis in WT and GhANT-1 via SEM, which may be primarily due to epidermis cells with a high rate of cell division but not of cell expansion (Figure 5C). We speculated that the enlargement of the ovules was caused by an increase in the cell number.

To clarify the effect of GhANT up-regulation on ovule growth, the expression profile of 18DPA ovules was analyzed in TFM7::GhANT and wild-type cotton plants. A total of 1652 DEGs were detected in GhANT; a further analysis indicated that numerous genes were up-regulated in TFM7::GhANT-1, including DNA replication licensing factor genes, six cell cycle control process-related genes, and 31 carbohydrate transport- and metabolism-associated genes. Moreover, we validated the RNA-Seq data by means of qRT-PCR (Figure 5B). The expression of GhANT was confirmed to increase in transgenic endosperm for Cyclin-A1-1-like protein and MCM, with a significant peak at 18 DPA. The cyclin proteins play important roles in the initiation and elongation steps in eukaryotic DNA replication, which occurs only once during the S phase of the cell cycle [37]. MCM (Gh_D05G2257) and CYCB1;1 (Gh_A02G1079) mRNAs were most abundant in cultured cells, shoot apices, and flower buds, which contain mitotic and endocycling cells [38].

These results strongly demonstrate that GhANT modulates cell cycle control to increase the seed size and seed yield in cotton. Therefore, we successfully obtained an understanding of the role of GhANT in controlling the seed size and yield of transgenic GhANT cotton, providing a promising tactic for cotton molecular breeding programs.

## Figures and Tables

**Table 1:** Yield quality of transgenic lines and wild-type in a three-year field trial. Different letters represent significant differences at *p* < 0.05 by one-way ANOVA with Tukey multiple comparisons test.

Year		Boll Number	Seed Number per Boll	Boll Weight (g)	Seed Index (g)	Lint Index (g)	Lint Percentage (%)	Upper Half Mean Length (cm)	Uniformity	Fiber Strength	Micronaire
2017	WT	21.98 ± 0.98	22.13 ± 1.64	3.72 ± 0.3 ^b^	10.13 ± 0.33 ^b^	6.49 ± 0.26	39.06 ± 1.43	27.57 ± 0.44	83.48 ± 0.31	29.83 ± 0.94 ^b^	5.4 ± 0.11 ^a^
	GhANT-1	21.08 ± 0.46	23.79 ± 0.35	4.33 ± 0.23 ^a^	11.28 ± 0.3 ^a^	6.68 ± 0.47	37.16 ± 1.07	27.78 ± 0.33	83.87 ± 0.39	30.83 ± 1.04 ^a^	5.17 ± 0.15 ^a^
	GhANT-2	22.67 ± 1.62	23.77 ± 0.84	4.39 ± 0.22 ^a^	11.21 ± 0.24 ^a^	6.84 ± 0.37	37.89 ± 1.72	28.10 ± 0.35	83.20 ± 0.52	32.82 ± 0.94 ^a^	4.83 ± 0.36 ^b^
2018	WT	23.12 ± 1.34	22.89 ± 1.12	3.74 ± 0.12 ^b^	10.13 ± 0.61 ^b^	6.34 ± 0.59 ^b^	38.64 ± 1.15	29.3 ± 0.31	86.43 ± 1.39	30.6 ± 1.91	4.78 ± 0.27
	GhANT-1	22.78 ± 2.13	23.36 ± 2.06	4.3 ± 0.24 ^a^	11.45 ± 0.66 ^a^	7.03 ± 0.34 ^a^	38.06 ± 0.76	29.57 ± 0.74	85.97 ± 0.69	30.95 ± 2.56	4.65 ± 0.41
	GhANT-2	23.58 ± 0.76	23.73 ± 1.33	4.33 ± 0.10 ^a^	11.33 ± 0.66 ^a^	6.94 ± 0.32	37.99 ± 0.77	29.43 ± 0.63	86.4 ± 0.57	30.55 ± 2.81	4.93 ± 0.16
2019	WT	23.45 ± 0.74	23.43 ± 0.44	3.55 ± 0.24 ^b^	9.75 ± 0.19 ^b^	6.47 ± 0.15	39.89 ± 0.54 ^a^	29.6 ± 0.37	85.07 ± 0.92	31.28 ± 1.48	4.72 ± 0.35
	GhANT-1	23.55 ± 0.30	23.65 ± 1.79	4.10 ± 0.09 ^a^	11.04 ± 0.29 ^a^	6.27 ± 36.22	36.22 ± 1.23 ^b^	29.61 ± 0.64	85.2 ± 0.6	31.5 ± 1.48	4.6 ± 0.21
	GhANT-2	23.12 ± 0.89	23.86 ± 1.26	4.26 ± 0.16	10.95 ± 0.33 ^a^	6.67 ± 0.18	37.86 ± 0.98 ^b^	29.7 ± 0.35	84.9 ± 0.17	30.53 ± 0.92	4.93 ± 0.19
Average	WT	22.85 ± 1.13	23.1 ± 0.89	3.67 ± 0.24 ^b^	10 ± 0.43 ^b^	6.43 ± 0.37	39.19 ± 1.17 ^a^	28.82 ± 0.99	84.99 ± 1.54	30.57 ± 1.53	4.97 ± 0.40
	GhANT-1	22.47 ± 1.55	22.15 ± 1.31	4.24 ± 0.22 ^a^	11.28 ± 0.46 ^a^	6.66 ± 0.47	37.15 ± 1.25 ^b^	28.98 ± 1.04	85.01 ± 1.04	31.09 ± 1.73	4.81 ± 0.37
	GhANT-2	23.13 ± 1.08	22.97 ± 1.04	4.33 ± 0.17 ^a^	11.16 ± 0.45 ^a^	6.82 ± 0.30	37.91 ± 1.16 ^b^	29.08 ± 0.84	84.83 ± 1.41	31.3 ± 2.01	4.90 ± 0.24

## Data Availability

The datasets generated during and/or analyzed during the current study are available from the corresponding author on reasonable request.

## Supplementary Materials

The following supporting information can be downloaded at: https://www.mdpi.com/article/10.3390/genes16080912/s1, Figure S1: qRT- PCR analysis the expression of GhANT in wild-type and GhANT-1 in different time. White represent WT and Black represent GhANT-1 transgenic line.; Figure S2: Southern blot analysis of WT and GhANT-transformed cotton lines. Genomic DNA was digested with HindIII. Figure S3: Phenotypic analysis of the transgenetic line GhANT-1 and wild type in the field. Figure S4: Phenotype analyses of the 35S-GhANT in Arabidopsis. (A) The Phenotype of the WT and 35S-GhANT transgenic Arabidopsis (40 day after sowing). Bar = 1 cm; (B) Phenotype of rosette leaves of the WT and 35S-GhANT transgenic plants; (C) Phenotype of silique the WT and 35S-GhANT transgenic plants. Bar = 1 mm. Table S1: Primer used in this study.
