# Supplementary material for: Special Regulation of GhANT in Ovules Increases the Size of Cotton Seeds
Source: Genes (Basel). 2025 Jul 30;16(8):912. doi: 10.3390/genes16080912 (PMC12385495; doi:10.3390/genes16080912)
Supplement: Supplementary file 1 [file genes-16-00912-s001.zip › genes-3740110-supplementary.pdf]

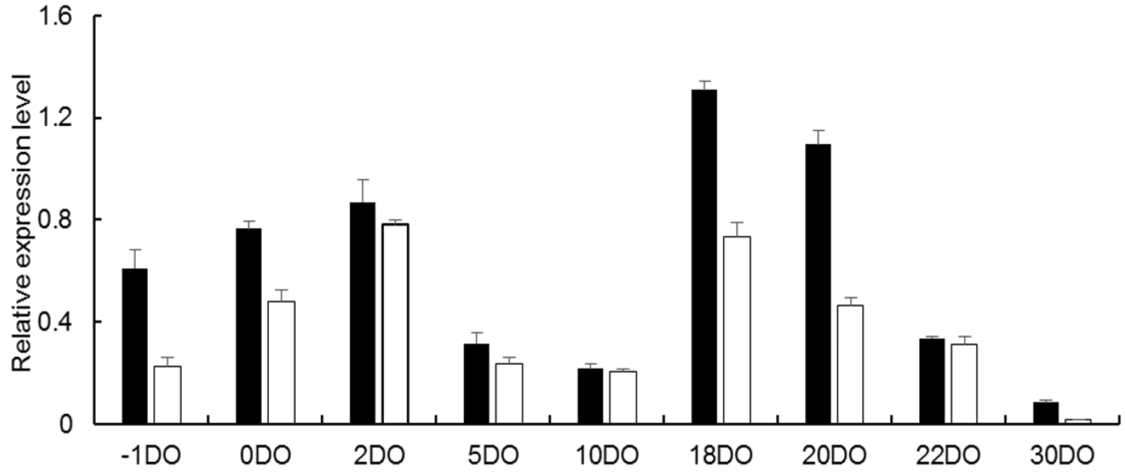

Figure S1. qRT- PCR analysis the expression of GhANT in wild-type and GhANT-1 in different time. White represent WT and Black represent GhANT-1 transgenic line.

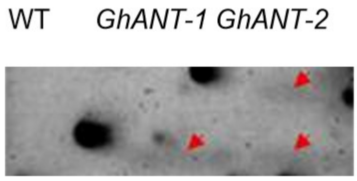

Figure S2. Southern blot analysis of WT and GhANT-transformed cotton lines. Genomic DNA was digested with HindIII.

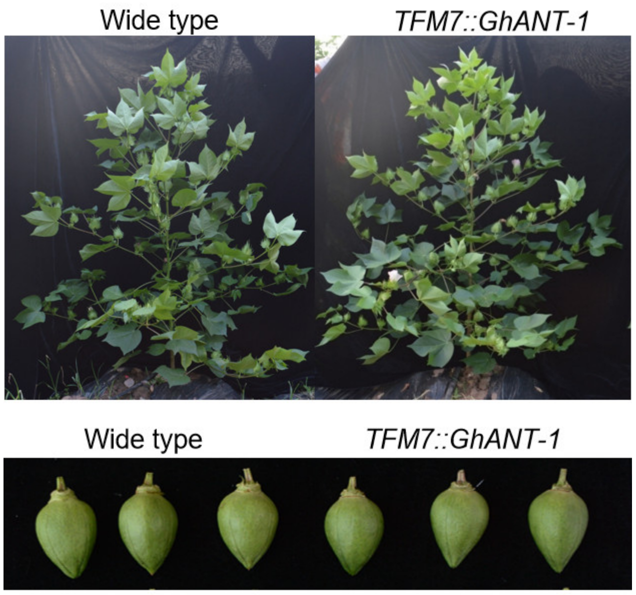

Figure S3. Phenotypic analysis of the transgenetic line GhANT-1 and wild type in the field.

 https://doi.org/10.3390/genes16080912

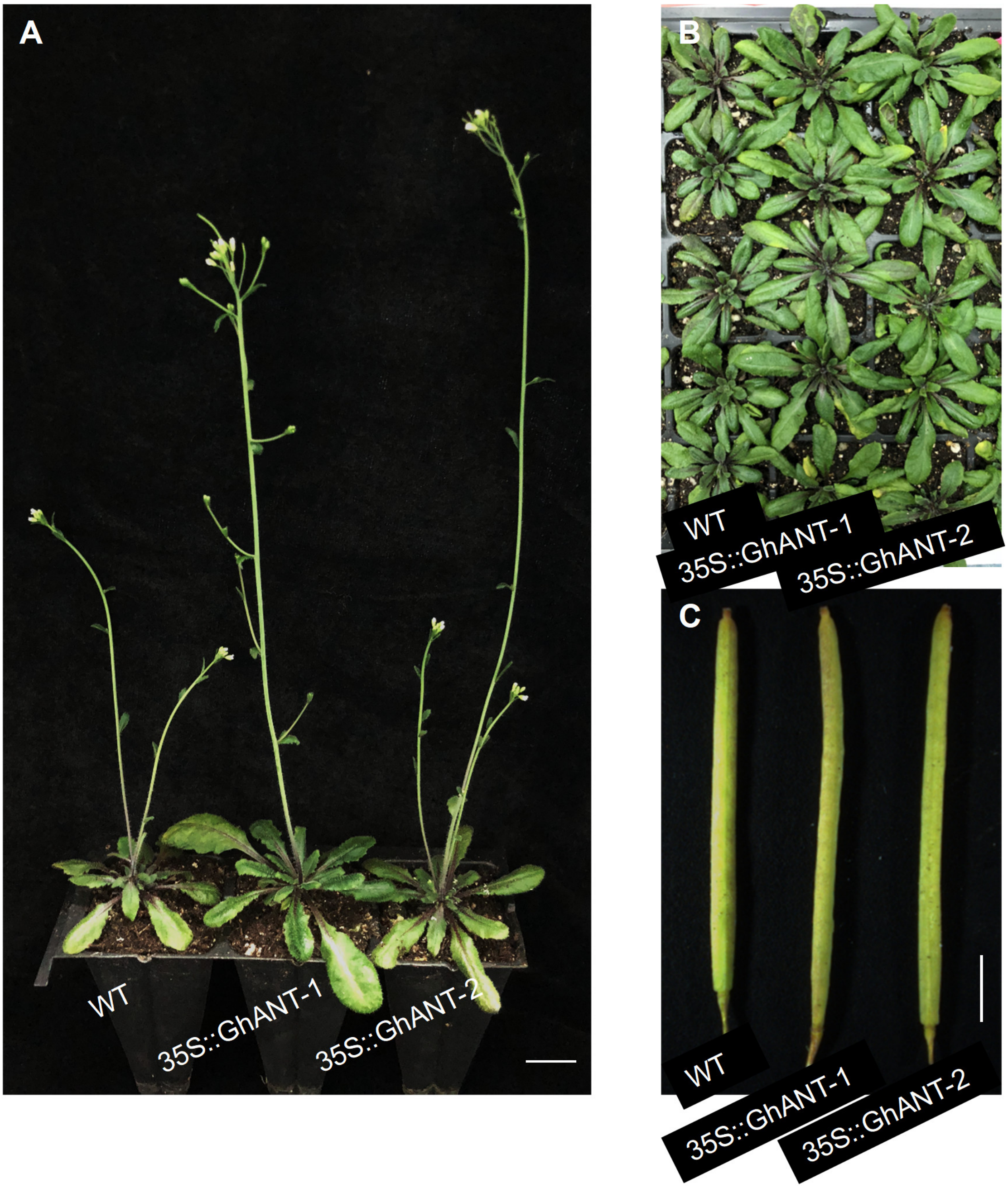

Figure S4. Phenotype analyses of the 35S-GhANT in *Arabidopsis*. (A) The Phenotype of the WT and 35S-GhANT transgenic Arabidopsis (40 day after sowing). Bar = 1 cm; (B) Phenotype of rosette leaves of the WT and 35S-GhANT transgenic plants; (C) Phenotype of silique the WT and 35S-GhANT transgenic plants. Bar = 1 mm.

Table S1. Primer in this study.

Primer name	Forward primer	Reverse primer
Histone3	GAAGCTGCAGAGGCATACC	CTACCACTACCATCATGGC
Actin	CCTGAGGTCCTTTTCCAACCA	GGATTCCGGCAGCTTCCATT
GhANTsense	CTTCTAATACGACTCACTATAGGGATTAGACGTTTTG GTATAGTAGC	GTCTTTAAAGTATTTCCCTCAATC
GhANTantisense	ATTAGACGTTTTGGTATAGTAGC	CTTCTAATACGACTCACTATAGGGGTCTTTAAAGTAT TTCCCTCAATC
pro10791LUC	CGGTATCGATAAGCTTGGAATGTGCAGCGGAACTGT	TAGAACTAGTGGATCCTCTAGCTCGTTATGCAATGG
pro2257LUC	CGGTATCGATAAGCTTGTGGATTAAATAGTAGAAGTG	TAGAACTAGTGGATCCCTCAGCTTTCTTTTATCCGAAAGAGAG
Gh_D11G1646	CCGATCAGTATTCCGGCCTC	GGACTGAACCAGGGCAATGA
Gh_A02G0669	GCAGTGGCAGAACTTGCATC	GAACCGTCGAGTGGATCGAA
Gh_A12G1844	GTAAATCTGACGGGAGGGGC	CCATCCGACAGCAATAGCCT
Gh_A04G1365	CTGGAAATCCCATCTCCCCG	AGTACCGCCAAGCTTCACTC
Gh_A09G0025	TCACGACAGCAGCCATTCAT	TCGTACCGGTCATGTGCATC
Gh_D11G1150	TCAAAGGGGAGGCCGTTTAC	TACAAGGGCAGGAATGCTCG
Gh_A03G1940	GATACAGGCGATGGAAGCGA	GGCGCTTGGTGAAAGGAATG
Gh_A03G0625	GCCTCCATCGGCTTAGGTTT	CTGGCATCCCTCACACCAAT
Gh_D02G2379	GATACAGGCGATGGAAGCGA	ATGACCGTGGGATTCTGCTC
Gh_D02G0837	GCGGACCACCGAGAAATACT	GACCCATGAGTCCCACATCG
Gh_D06G0684	ATGCTGCTCATGTTGAGGCT	GTCCTGCACCCTCTGCTATC
Gh_D09G1575	TCAAGGTTCCGTTTACCCCG	AATGTGACAACGCAAGCACC
pTFM7 primer	gggcaatgaacaaagttcca	agctttgcatcccaaatag
